# Pet primates for sale in the United States

**DOI:** 10.1371/journal.pone.0256552

**Published:** 2021-09-08

**Authors:** Melissa S. Seaboch, Sydney N. Cahoon

**Affiliations:** 1 Anthropology Department, Salt Lake Community College, Salt Lake City, UT, United States of America; 2 Anthropology Department, University of Utah, Salt Lake City, UT, United States of America; University of Lincoln, UNITED KINGDOM

## Abstract

Our research goal was to investigate the primate pet trade in the United States. While dogs and cats are the most common type of pet, there are an estimated 15,000 pet primates in the United States and the demand for exotic pets in general has been rising. Most research on pet primates occurs in habitat countries and little is known about these pets in the United States. We collected data from six exotic pet-trade websites twice a month for 12 months. We recorded the type of primate for sale, sex, age, location, and price. We used Chi-Square Goodness-of-Fit tests to compare whether the number of male and female pet primates for sale and the number of different age categories of pet primates for sale differed from equality and Spearman Correlation to examine associations between price and size and price and supply. We recorded 551 pet primates for sale between June 2019-June 2020, with 69.1% platyrrhines, 21.6% strepsirrhines, and 8.9% catarrhines. Marmosets were sold most often (36.7%, N = 202) followed by lemurs (21.6%, N = 119), capuchins (11.3%, N = 62), and squirrel monkeys (10.5%, N = 58). Almost two-thirds of the pet primates for sale were male (Chi-Square = 16.056, df = 1, P = 0. 00006) and 78.7% were under one year old (Chi-Square = 440.264, df = 2, P<0.00001). The median price was $3,800 though price was highly variable, even for the same taxa. There are several potential drivers for the primate pet trade, including media influence, fashion/status, and profitable breeding though these are not mutually exclusive. Primates do not make good pets and even when captive-bred, pet primates impact the conservation of their wild counterparts. Advertisement campaigns focusing on disease transmission and legal consequences and a federal ban on pet primate ownership are two avenues to pursue to end the ownership of pet primates in the United States.

## Introduction

Recent surveys of pet owners have found that human attachment to their pets (animals kept within a domestic setting for personal interest, entertainment, or companionship [1]) plays an important role in their lives as evidenced by how they refer to their pets–as friends, children or fur babies, and members of the family. Owners often attribute human qualities to their pets describing them as thinking, emotional, and creative [2–5]. Young adults in distress may be more likely to turn to their dogs than to some family members [2]. More than half of pet owners report they are closer to their pet than to their own parents and 95% of dog owners hug their companions every day [2].

Humans have owned pets for many millennia. Evidence suggests that the first pet, the dog, was domesticated by 30,000 years ago, likely for practical purposes such as tracking and hunting [6]. Archaeological evidence of a social bond between dogs and people, as companions, is found by 14,000 years ago [7,8] suggesting that people have valued animal companions for thousands of years. Evidence of nonhuman primates (hereafter referred to as primates) as pets is similarly ancient. In an Iranian cemetery dating to 4,800 years ago, a rhesus macaque (*Macaca mulatta*) was buried with grave goods and in the same manner as the human children in the cemetery [9]. The macaque had pathologies of the hindlimbs indicating that it was inadequately cared for (possibly kept in a cage too small to allow it much movement) and had been imported as macaques are not endemic to Iran [9].

Approximately two-thirds of all households in the United States include pets [2,10]. Much research has touted the benefits of owning a pet to one’s physical and mental health [8,11]. Additionally, specially trained pets can provide service to their owners, and even save their lives (e.g. Medical Alert Dogs (MADs) [12]). People have a strong social and emotional attachment to their pets [11]. When asked why they own pets, reasons included having companionship, having a play partner, and the need to love and care for another creature and most pet owners believe their pets are good for them [3,10].

Though dogs and cats are the most common type of pet, primates are also kept as pets. It is estimated that there are over 15,000 primates owned as pets in the United States [13], which is a small, but not insignificant number. This is despite the almost universal opinion of scientists and veterinarians that primates do not make good pets; primate pet ownership is detrimental to the primates themselves as well as to their human owners [14–18]. Many primate pet owners do not have a sufficient understanding of their species or of how to care for them which can result in nutritional deficiencies, injuries, and behavioral disorders [13]. Primates are naturally aggressive and injuries to their owners are not uncommon [5,13]. Also, several diseases can be transmitted from primates to humans (e.g. parasites, Salmonella, rabies) [13,19].

Owning an exotic pet is an increasingly larger part of the wildlife trade network, it is the third largest illegal trade [20–22], and monkeys are becoming “ever more fashionable” as pets [20, p. 2408]. A study of the global trade in exotic pets found that primates and carnivores were the most often traded mammals, though mammals were less common than birds and reptiles [1]. This study also found that primates were rarely traded at large physical markets. Research on pet primates has been increasing, though most studies focus on pet primates in habitat countries or countries near habitat countries that themselves have endemic primates (see, for example, Reuter et al., [23] for Madagascar, Duarte-Quiroga & Estrada [24] for Mexico; Ceballos-Mago et al., [25] for Venezuela; Nijman et al., [26] for Indonesia). Systematic studies examining the primate pet trade in the United States are rare. The goal of this research is to preliminarily investigate the pet primate trade, including which taxa are for sale in the United States, the sex and age of the primates for sale, the seller’s location, and the price to provide a baseline for future research.

## Methods

### Ethical research considerations

Though we were collecting publicly available information from the surface web, the research was also approved by an ethics oversight committee (Institutional Review Board, University of Utah) in 2019 as an amendment to IRB_00079146. The complete dataset is stored on an encrypted hard drive and all personal identifiers were removed from the analysis.

Data were collected manually twice a month for 12 months (June 2019-June 2020) from six publicly available online exotic pet-trade websites (their identifications are not being included to avoid unintentionally raising their visibility). Prior research has suggested that data collected from the Internet can provide a reliable indicator of the exotic animal pet trade globally [27,28] and can be useful in describing some aspects of the exotic animal trade. Additionally, the use of the Internet in the trade and trafficking of exotic animals has been increasing [20–22,28–32]. Though primates are for sale through a variety of venues, publicly available exotic pet sale websites were selected because data are less personalized than social media, such as Facebook or Instagram, thus reducing ethical issues with data collection. For example, some Facebook groups require that you join which involves a certain level of deceit. E-commerce websites were located through a search engine (Google) using the phrases “monkey(s) for sale United States” and “primates(s) for sale United States” and we examined the first 50 results from each search for relevance [28]. Search results that did not return e-commerce websites showing primates for sale (e.g., websites discussing the appropriateness of pet primates, forums, blogs), local classified advertising websites, such as city-specific websites, and websites that did not list specific primates for sale (e.g., websites that only included phrases such as “contact … for availability”) were excluded. From the remaining results, we selected the six most popular sites, as indicated by their ranking in the search engine results, that had the most primates for sale in our initial review. These six websites were then searched for “monkey”, “primate”, “lemur”, “loris”, “galago”, “bushbaby”, “baboon” and “ape”. We recognize that our keyword search was limited. However, more extensive searches were done for the first two months in our data collection process (e.g., broadening our keyword search for specific types of primates, specifically those listed in state regulations, such as “tarsier”, “prosimian”, “indri”, “sifaka”, “marmoset”, “tamarin”, “capuchin”, “saki”, “uakari”, “muriqui”, “guenon”, “langur”, “macaque”, “mangabey”, “mandrill”, “drill”, “gibbon”, “chimp”, “gorilla”, and “orang”), but these searches did not yield any results not initially found by our primary search terms, thus we limited the remainder of our keyword searches for efficiency.

For each advertisement, we recorded the date the advertisement was posted, seller’s username, common name of the primate, sex, age, location, and price. Very few advertisements listed a genus or species and most descriptions used common names (e.g. marmoset, capuchin) so we aggregated our data by these general types. We also downloaded photos of the primates for sale. The seller’s username and photos were recorded to avoid re-counting the same advertisement if it was posted for multiple weeks or on multiple websites [28]. Advertisements that were not clearly for a specific primate for sale (e.g. some advertisements stated “contact us to see what primates we currently have”) were not recorded in our dataset. Additionally, animals that were clearly misidentified (e.g. listed as capuchin, but photos in advertisement were clearly cercopithecine) were excluded from the analysis; two advertisements were removed for this reason. To prevent inflating the number of primates for sale, we recorded the number of primates for sale as one regardless of the number of primates in photos provided in the advertisement and despite the use of plural descriptions (e.g. monkeys) unless the advertisement specifically noted they had more than one primate for sale (e.g. if an advertisement stated three primates were for sale, then we recorded that as three primates). Advertisements that stated that there were “several” or “multiple” or “many” primates for sale were recorded as having two primates for sale.

We were unable to verify the validity of these online advertisements and we are aware that pet scams exist, that they had been increasing, but that there was a decline in 2020 [28,33,34]. To reduce the impact of scams, we examined the accompanying photos as an indicator that the animal was in the possession of the seller [33,35,36] and compared photos on different websites because posting the same advertisement can be an indicator of a scam [33,34,36]. Also, five of our six websites have been in existence for over 10 years. Scams often offer pets for free or at a deeply discounted rate, sometimes mentioning that the animal must be sold because of a family hardship [33,34,36]; this tactic was not present in any of the advertisements we recorded. Also, Price [34] found that scam websites often targeted a single taxon while our websites listed a wide variety of taxa (e.g. mammals, birds, reptiles). Finally, we searched Pet Scams (petscams.com), which catalogs pet scam websites, and Scam Detector (scam-detector.com), an official contributor to the Federal Trade Commission which uses a complex algorithm to yield a “trust index”, for all six of our websites. None of our websites were included in the Pet Scams catalog and the Scam Detector “trust index” ranged from 55.7 to 75.8 (100 is the most reputable); the websites that scored below 70 did so because the Scam Detector algorithm determined their website was poorly designed.

Sex and age were compared using a Chi-Square Goodness-of-Fit test with the null hypothesis that the number of males and females would be equal and that the number of primates for sale in different age class would be equal. Ages were binned into three categories (< 1 year, 1–2 years, > 2 years) because animals were often identified with descriptors (e.g. “baby”) rather than by chronological age. Primates listed as “baby” were included in the < 1-year category while primates listed as “adult” were included in the >2 year category, though we recognize this binning of ages does not take into account the life history of the species (i.e. different species reach adulthood at different ages). Range, mean, median, and mode were calculated for price. Spearman’s Correlation (α = .05) was used to test for an association between the median price per taxa with pooled mean adult body size (mass) [37] and with the number of primates for sale (“supply”).

## Results

**Fig 1:**
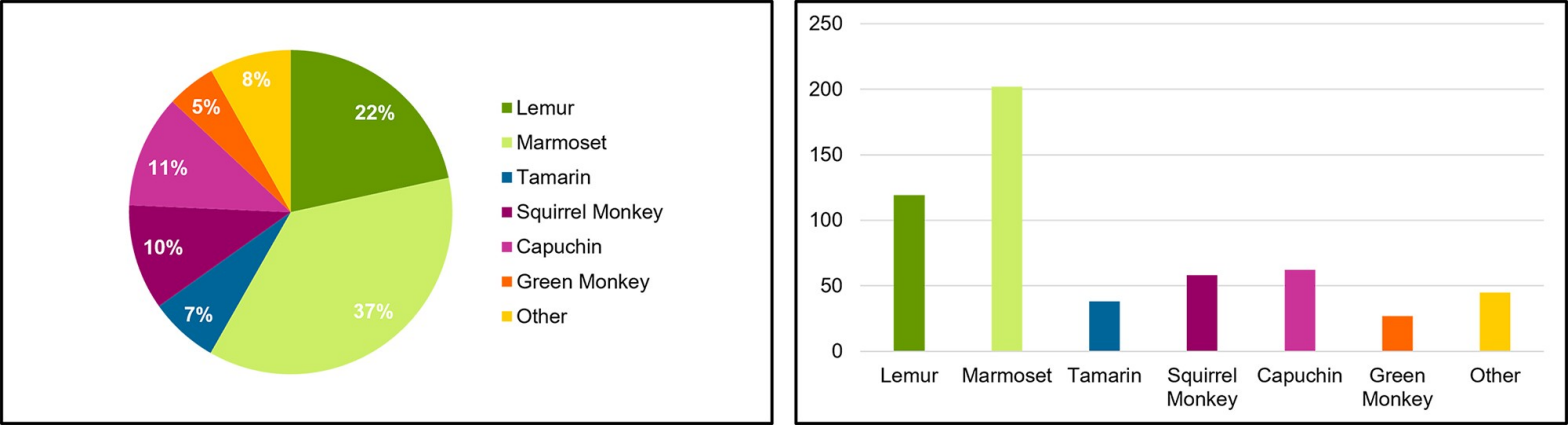
a and b. Primates for sale in the United States. Proportion of pet primates for sale. Number of Primates for sale.

**Fig 2:**
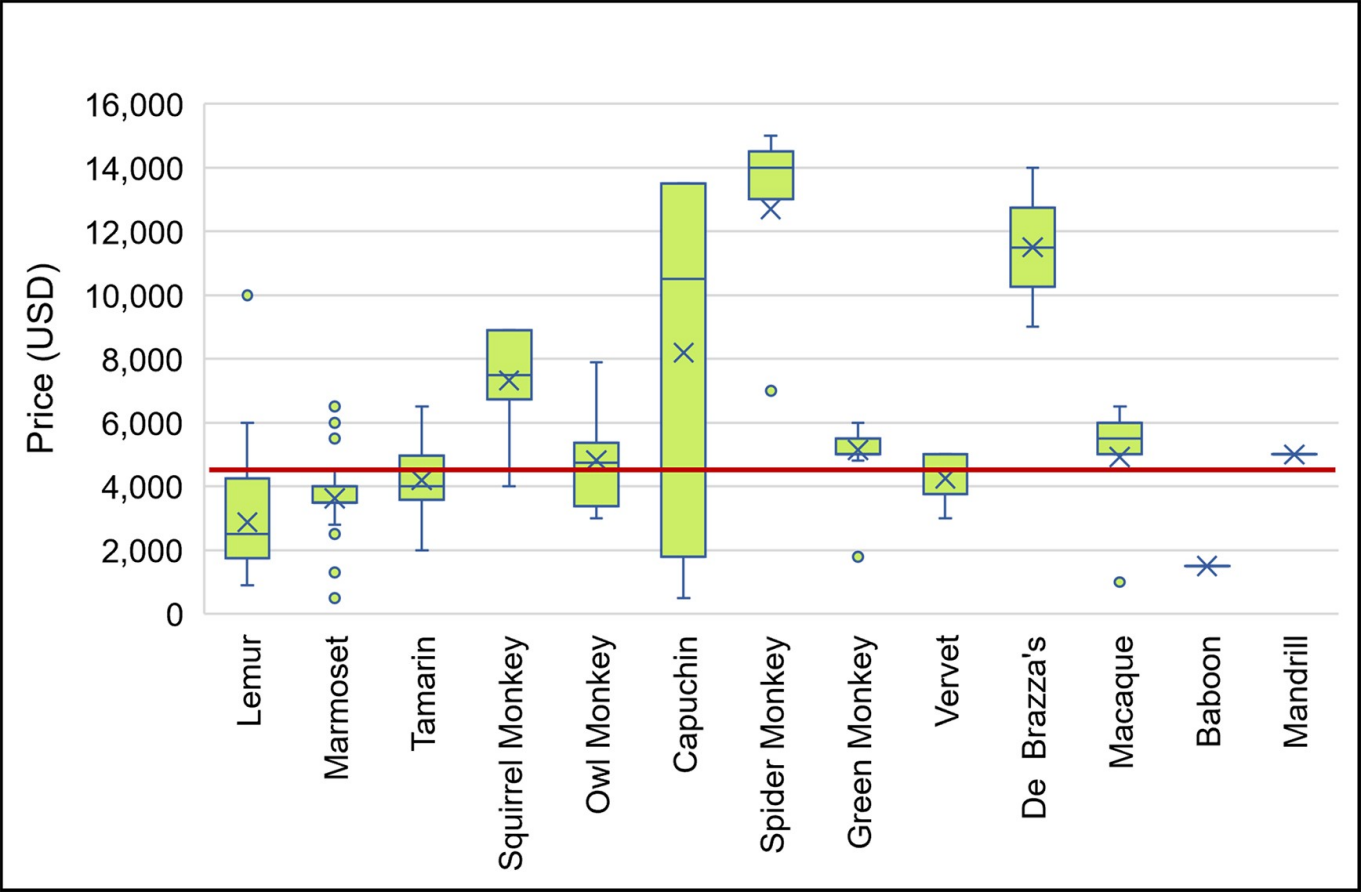
Price of primates for sale in the United States. Red line represents sample average ($4,618.14).

We identified 551 primates of 14 different taxa for sale (Table 1). Marmosets were the most common taxon of primate for sale (36.7%, N = 202) followed by lemurs (21.6%, N = 119), capuchins (11.3%, N = 62), and squirrel monkeys (10.5%, N = 58 (Fig 1A and 1B). We also found one baboon and one mandrill, both male, for sale. The majority (69.1%, n = 381) were platyrrhines, 21.6% were strepsirrhines (lemurs were the only strepsirrhines for sale), and only 8.9% (N = 49) were catarrhines with green monkeys being the most common catarrhine (4.9% of total primates for sale, 55% of catarrhines for sale, N = 27). Of the 288 advertisements which identified sex of the primate, 61.8% were male (N = 178), and 38.2% were female (N = 110) (Chi-Square = 16.056, df = 1, P = 0.00006). Of the 467 advertisements listing primate’s age, there were more primates under 1 year for sale (78.6%, N = 367), compared to 1–2 years (4.7%, N = 22) and older than 2 years (16.7%, N = 78) (Chi-Square = 440.264, df = 2, P<0.00001). Primates were found for sale in 22 states. Florida had the most primates for sale (45.7%, n = 252), followed by Tennessee (11.8%, n = 65), Texas (11.6%, n = 64), Missouri (6.7%, n = 37), and North Carolina (5.6%, n = 31). The price of the primates ranged from $500 for a capuchin or marmoset to $15,000 for a spider monkey with a mean of $4,618.14 ± SD $2931.20, a median of $3,800, and a mode of $3,500. Price varied widely within and between taxa (Fig 2). For example, prices for capuchins ranged from $500 to $13,500. Average price per taxa was not correlated with the size (r_s_ = 0.2531, p = 0.40411) or supply (r_s_ = -0.11279, p = 0.71371) of the primates. The two largest primates (baboon and mandrill) were near or below the sample average (baboon = $1,500 and mandrill = $5,000) while marmosets, the smallest primates, were just under the sample average (marmoset average = $3,624.31).

**Table 1:** Sampling of primates for sale in the United States.

Primate	Total	Sex		Age Category		
		Male	Female	< 1 Year	1–2 Years	> 2 years
Baboon	1	1	0	0	0	1
Capuchin	62	10	14	39	1	9
De Brazza’s Monkey	3	1	1	2	0	1
Green Monkey	27	7	9	25	0	1
Lemur	119	50	27	54	6	33
Macaque	9	5	3	4	2	2
Mandrill	1	1	0	0	0	1
Marmoset	202	58	34	160	9	10
Owl Monkey	11	5	3	6	0	3
Patas Monkey	1	1	0	1	0	0
Spider Monkey	10	7	3	9	0	1
Squirrel Monkey	58	18	5	44	1	7
Tamarin	38	8	8	19	2	5
Vervet	7	4	3	2	1	4
Unidentified	2	2	0	2	0	0
Total	551	178	110	367	22	78

## Discussion

Our finding of 551 pet primates for sale over the course of one year underestimates the true numbers because we only examined six exotic pet websites advertising primates for sale in the United States. However, primates are for sale through numerous other venues including private and commercial breeders, auctions, social media, and even pet stores [28].

We had no *a priori* hypotheses for what types of primates would be for sale in the United States. Prior research on pet primates generally occurs in habitat countries with wild-captured animals (see, for example, [23–26]) and, therefore, provided little foundational background for the U.S. market of captive-bred animals. However, several possibilities for the drivers of the primate pet trade in the United States exist. In a market-driven system, buyers (demand) are likely influencing the sellers (supply). Perceived “ease” of care and/or the depiction of primates in the media may influence the type of primate that buyers desire. Additionally, the owning of a pet primate in general (as opposed to owning a specific type of primate) may be fashionable or a symbol of status. Most profitable breeding practices may be a driving factor for the sellers. These possibilities are not mutually exclusive.

### Potential drivers of the primate pet trade in the United States

#### Popularity of primate taxa in the media

The desire to own exotic pets may stem from the influence of their popularity in television shows and films. Studies have shown that the sale of green iguanas increased after Jurassic Park [38] and the sale of red-eared slider turtles increased after the release of Teenage Mutant Ninja Turtles [39]. Similarly, the sale of clown fish increased after the release of Finding Nemo [40] and the sale of owls increased after the Harry Potter movie franchise premiered [41] (though see [42]).

Similar results have been reported for common pets. For example, the movie Snow Dogs was associated with an increased popularity of Siberian huskies and border collies [43]. Several releases of, or sequels to, 101 Dalmatians were associated with trends in the increase of dalmatian sales [44] and Jack Russel Terriers became trendy with the TV show Frasier [45]. This trend goes back several decades as Old English Sheepdogs became popular with the release of the Shaggy DA films in the 1960s [44].

Primates have been featured in films since the 1930s [46]. Aldrich [46] found that between 1990 and 2013 chimpanzee were the most common primate “actor” (42% of films). While Fleury [47] asserted in 2013 that chimpanzees were the most common primate pet in the United States, the Chimp Care website [48] indicates there are currently 25 chimpanzee pets in the United States and we found no chimps for sale in our study. In 2015, the Fish and Wildlife Service (FWS) Rule 80 FR 34499 classifying captive chimpanzees as “endangered” (effectively ending the private legal ownership of chimpanzees by requiring permits, granted only for scientific research, [49]) probably drove this reduction in the number of pet chimpanzees. Though we note that it is illegal in most states to own a chimpanzee [50], we did find other illegal primates for sale (e.g. a mandrill was advertised for sale in Florida even though they are barred from personal possession through the Captive Wildlife Licenses and Permits Rule 68A-6.002 of the Florida Fish and Wildlife Conservation Commission and eight primates were for sale in three states, Colorado, Georgia, and New York, where ownership is banned [50]).

According to Aldrich [46] the other primate “actors” were capuchins (33%), cercopithecines (macaques and baboons) (13%), orangutans (8%) lemurs (2%), and gorillas (2%). One capuchin, Crystal, is credited in 26 films [51] including George of the Jungle, the Doctor Doolittle films, the Night at the Museum films, and the Hangover films. In our study, we found no gorillas or orangutans for sale, only one baboon, and nine macaques. Our results for capuchins and lemurs provide limited support for the idea that popularity in film and television may be a driver for the choice of primate for a pet. Capuchins, the third most common primates for sale, have been featured regularly in films and TV shows and, if including animated shows such as the Madagascar franchise, so have lemurs, the second most common primate for sale. Adults who were raised watching Zoboomafoo or the Madagascar franchise may see a sifaka or a ring-tailed lemur as a potential pet and not as a wild animal. In interviews with primate pet owners, some speak of how their childhood love of books and movies depicting primates spurred them to purchase primate pets in adulthood [5]. According to Aldrich [46], no marmosets, the most common type of primates for sale, were featured in films between 1990 and 2013. As this study did not survey owners, it is not known whether these choices in pets were related to their depictions in film and/or on television.

#### Popularity of primates in general

While the main goal of this project was to uncover which types of primates were for sale, future research addressing why primates are for sale as pets in the first place is needed. While many of the advertisements we examined described the primates for sale in favorable terms, such as “cute”, “cuddly”, “friendly”, “sweet”, “adorable”, “loving”, and “tame”, most scientists and veterinarians agree that primates do not make good pets because they are aggressive, they are difficult to properly care for, and they can transmit diseases [13–18]. Recent research on preferred dog breeds suggests that fashion, and not good sense, can drive the selection of specific breeds. In the United Kingdom, each of the top 50 dog breeds had at least one inherited disorder [52]. Additionally, dog breeds are not selected based on good health, longevity or good behavior (i.e. the most popular dogs are often those with high frequencies of genetic disorders and poor behavior) [53]. Similarly, owning a pet primate may not be based on good sense and it is not uncommon for primate owners to admit that they did not realize what they were getting into when deciding to get a primate describing their pets as “risky”, “unpredictable”, and dangerous” and that caring for the primates is “labor intensive”, “totally consuming”, “expensive”, “You never know which ones will grow up and attack”, “It’s sad to watch the depression they go through if they’re not getting enough one-on-one attention”, and “If given the chance to turn back the hands of time, … ‘I wouldn’t have a pet monkey’” [5,54].

Relatedly, primate ownership, like other exotic and more common pets, may be related to status [3,4,55]. The average cost of a pet primate in our study, around $4,000, is significantly more than the average cost of a dog or a cat. Purchasing such an expensive pet may serve to advertise the owner’s economic class and is an example of conspicuous consumption; it provides a boost to the owner’s ego [55]. Additionally, primate pet owners, along with owners of other exotic pets, have described several reasons for wanting to own something other than a dog or cat, including that it’s “cute”, it’s “cool”, to “show off”, to “impress others”, and as a way to “get attention” [5,56,57].

Social media could increase the desire for a pet primate [58,59]. Several famous individuals have posted selfies with pet primates. For example, Justin Bieber and Chris Brown owned pet capuchins, while Kristie Alley owned pet lemurs and Michael Jackson owned a pet chimpanzee named Bubbles [57,60]. Other celebrities, while not owning pet primates, have posted selfies with primates. For instance, Rihanna posted a selfie with a loris and Paris Hilton posted selfies with several different primates, including a capuchin and an orangutan [60–62]. Non-traditional celebrities, such as bloggers, YouTube personalities and Instafamous, are even more influential [63]. Kelvin Peña, otherwise known as Brother Nature, has posted selfies on Instagram with capuchins and a baboon at a Los Angeles sanctuary. He has also posted selfies on both his Instagram and Twitter with a cotton top tamarin and a ring-tailed lemur inside his home, making it likely they are his pets. Brother Nature has roughly 2.4 million followers. On TikTok, @heresyourmonkeycontent has almost a million followers and frequently posts video content of their tufted capuchin. Some primates are social media celebrities in their own right. For example, Pizzatoru, is a galago with an Instagram account and has 240,000 followers. Comments posted in response to photos of primates often express a desire to own one [58,59]. Photos of celebrities with primates can be especially problematic because many consumers copy celebrities to enhance their own self-esteem [64].

### Size, sex, and age of primates for sale

Size may be a driving factor in the choice of pet primates as smaller primates are easier to care for (e.g. smaller primates require less food, less space) and manage/handle than larger ones. They require less space and less food and may be easier to handle than larger primates. The adult size of the types of primates for sale range from 0.3 kg (marmosets) to 31.6 kg (male mandrill) [37]. In our study, 77% of the primates for sale were under 2 kg as adults and 95% were under 5 kg. A preference for small animal pets has also, but not universally, been reported for dogs [65–68]. Posage et al., [67] posit that a preference for small dogs could be because small dogs are easier to control, an idea supported by surveys of dog owners [69], and which would also be true for pet primates. Also, small versions of other pets, such as Munchkin cats, miniature pigs, and even miniature cows have become more popular [70–73]. Despite the high percentage of smaller primates, we found listed for sale, primates do not make good pets no matter the size, as noted by veterinarians and scientists [13–19]. While size is likely a driver in the choice of primate to buy and to sell, other factors are probably also at play.

Smaller primates tend to reach adulthood and begin reproducing earlier and have shorter gestation periods and interbirth intervals compared to larger primates. This faster life history can increase their reproductive success; thus, they can produce more offspring over a shorter period of time [74]. This is especially true of marmosets, the most common type of primate for sale (37% of advertisements). Marmosets regularly produce twins, with triplets also being common in captive colonies [75] and, with post-partum estrus, they can produce two litters in a single year [75,76]. In fact, marmosets and tamarins have the highest potential fecundity and fertility of any haplorrhine primate [76]. In one study, captive *Callithrix jacchus* produced an average of 3.66 offspring per year and had an interbirth interval of 217 days [76]. Consequently, smaller primates, especially marmosets, may be preferred by buyers because they are perceived as easier to care for than larger primates, and they may be preferred by sellers because of their higher reproductive output.

Breeding practices to maximize reproductive output may also account for the sex-bias in the primates for sale. In our sample, two-thirds of the primates for sale were male. Among breeders of other animals, females make up the largest portion of the breeding establishment (see, for example, [77,78]). Reproductive efficiency is not highly impacted by the number of males owned, but by the number of females; in other words, a breeder only needs a limited number of males to produce the maximum number of offspring. This could lead to breeders keeping more female offspring and selling more male offspring.

The sale of infants over older animals is unsurprising as puppies and kittens are also overwhelmingly preferred as pets [22,65,66,79–83]. Also, selling the offspring as infants reduces the amount of care and resources that must be invested by the breeder, thereby increasing profits. These profits would be compounded as the mothers, once infants have been weaned, can begin producing their next litter. Additionally, younger primates are less aggressive having not yet reached sexual maturity [13,24,84].

### Conservation implications

The primates we found for sale are presumably from breeders and not wild-caught [13]. It has generally been illegal to import primates since the 1975 Convention on International Trade in Endangered Species of Wild Fauna and Flora (CITES) entered in force. Also, a study of the global exotic pet trade found limited trade from primate-habitat continents (e.g. Central and South America, Africa) to North America [1] (though see [85] whose analysis suggests substantial export of squirrel monkeys and capuchins from Central and South America). Nonetheless, primates in captivity impact the conservation of their wild counterpart [86–88].

Research has shown that when people see primates outside of their natural habitat, it can increase their desire to own a primate themselves (which can drive the extraction of primates from the wild in habitat countries), lead them to believe they are not endangered, and decreases the likelihood they will contribute to conservation [58,59,89,90]; all of these will impact wild populations. Ross and colleagues [89,90] report that 35% of respondents did not know chimpanzees were endangered because of their frequent presence in films and television shows. Squirrel monkeys, capuchins, and ring-tailed lemurs were also seen as acceptable pets to individuals who have seen them depicted in close contact with humans out of their natural environment [91]. Primate pet owners may also think that by owning and/or breeding primates, they are saving them from extinction [92]. As Reuter & LaFleur [88] note, because of the global connectedness through social media, every primate kept as a pet is driving, either directly or indirectly, the capture of primates in the wild and is, therefore, impacting their conservation. Unfortunately, a study by Moorhouse et al., [87] found that informing a potential owner about an exotic species’ conservation status did not impact the desire to own one. In fact, rarity of a species (i.e. those more highly endangered) may have the opposite effect and increase their attractiveness as a pet [20,87].

### Reducing the pet primate trade

With the estimated high number of primates as pets in the United States and the seemingly healthy market of primates for sale as pets, this is a problem that will continue. There are several avenues for reducing the number of pet primates, including educational advertisement campaigns and legislation [22,86].

One path towards reducing pet primate ownership is targeted advertising campaigns to educate those considering purchasing a primate. Moorhouse et al., [87] found that advertisement campaigns focusing on disease transmission and legal consequences could reduce the demand for exotic pets by 39% while information on their conservation status or welfare would not reduce the demand at all. In short, potential owners of exotic pets were swayed by information on how owning an exotic pet would affect them, but not by information on how it would affect the pet primate. This study provides sound guidance for those seeking to educate the general public about the unsuitability of primates as pets.

Secondly, Beetz [86] proposes that a tax on exotic pets could limit the pet trade through simple economics. Similar sin taxes have been successful in changing behavior in other areas such as cigarette and alcohol use and the consumption of unhealthy foods and drinks (see, for example [93–96]). A recent study on cigarette usage found that state taxation, federal taxation, and an anti-smoking advertising campaign all significantly reduced the purchase of cigarettes, but the taxation reduced cigarette consumption more than the advertising campaign [97]. Since primates are so expensive to begin with, the tax would have to be high to make an impact. Additionally, for those purchasing primates as a sign of status, it is unlikely that taxation would impact their decision.

Another path involves federal regulation of the pet primate trade [22]. Currently, there are no federal regulation on the ownership of pet primates, though bills, such as s H.R.3135/S.1588 –Captive Primate Safety Act—which would ban the interstate trade and private ownership of primates, have been introduced in Congress multiple times, most recently in May 2021 [98], but not yet passed. Instead, the regulation of the primate pet trade is handled state by state creating a patchwork of laws. Federal legislation requiring permits to own primate pets may be another route to decrease their ownership if ownership cannot be banned outright. The recent decrease in the number of pet chimpanzees following the implementation of permit requirements for all captive chimpanzees (see above) suggests this may be a viable option.

## Conclusion

Our study of six exotic pet trade websites uncovered over 500 primates for sale over a one year period. This despite the belief of primatologists and veterinarians that primates should not be owned as pets [1–18]. They can transmit diseases to their owners and they can be aggressive, especially after reaching sexual maturity. Also, they are difficult to care for properly [13]. Most primates were for sale in Florida and were small-bodied primates, male, and infants; this is unsurprising as it would be most profitable for the sellers focus on smaller primates with their faster life history and to sell young primates and keep female primates for breeding.

While the pet primates for sale are likely captive bred, they can still have consequences for their wild counterparts. Making the ownership of pet primates illegal through federal regulation would likely reduce (but not eliminate) the number of pet primates both by making the purchase of one more difficult and by fear of legal consequences (as found by Moorhouse [87]). Research on primate pet owners themselves, including why they decided to own a primate, what drove their decision in the type of primate to purchase, and how the primate was purchased are urgently needed because understanding the demand for pet primates is the first step in reducing the demand [99].

## Supporting information

S1 File Data on pet primates in the United States. (XLSX)
